# An Absorption and Plasma Kinetics Study of Monoterpenes Present in Mastiha Oil in Humans

**DOI:** 10.3390/foods9081019

**Published:** 2020-07-30

**Authors:** Efstathia Papada, Aristea Gioxari, Charalampia Amerikanou, Nikolaos Galanis, Andriana C. Kaliora

**Affiliations:** Department of Dietetics and Nutritional Science, School of Health Science and Education, Harokopio University, 70 El. Venizelou Ave, 17671 Athens, Greece; efpapada@gmail.com (E.P.); arisgiox@gmail.com (A.G.); amerikanou@windowslive.com (C.A.); ngalanis@gmail.com (N.G.)

**Keywords:** monoterpenes, mastiha oil, absorption

## Abstract

Monoterpenes are bioactive compounds, however studies on their metabolic fate in humans are scarce. The present work aimed to identify and quantify the bioactive monoterpenes myrcene, α- and β-pinene of the Mediterranean product Mastiha Oil, in human plasma after acute consumption of a single dose. This was an open-label, single-arm acute study. After overnight fasting, healthy males were administered with Mastiha Oil. Blood samples were collected on different time-points before and after consumption. A novel GC-MS-MS application was performed to detect and quantify terpenes in MO and in plasma. Serum lipid resistance to oxidation was also determined. Alpha-Pinene, β-pinene and myrcene were identified and quantified in plasma. Alpha-pinene concentration significantly increased after 0.5 h of Mastiha Oil consumption, remaining significantly increased at 1 h, 2 h, 4 h, 6 h and 24 h. Beta-pinene and myrcene followed similar patterns. The increase in serum lipid resistance to oxidation was significant at 1 h, reached its peak at 2 h and remained significant until 4 h. Conclusively, α-pinene, β-pinene and myrcene that are present in Mastiha Oil are absorbed by humans. (ClinicalTrials.gov Identifier: NCT04290312).

## 1. Introduction

Natural and plant-derived products or additives contain large amounts of non-nutrient phytochemicals. There is abundant research upon their effects on health, with several studies revealing beneficial properties on the prevention of chronic diseases, mainly through the activation of the immune system [1]. Nevertheless, evidence on the fraction of administered compounds that can pass into the plasma and body tissues without changing their structure, is yet very limited and often zero. This step is necessary to understand bioactivity and to prove efficacy. 

Mastiha Oil (MO) is extracted from the resin of Mastiha, the dried resinous exudate from stems and branches of Pistacia lentiscus. Mastiha is a concentrated source of monoterpenes (e.g., α-pinene, β-pinene, β-myrcene) [2], triterpenes (e.g., mastihadienonic acid, isomastihadienonic acid) [3], and to a smaller degree of plant sterols, simple phenols and approximately 10% MO [4]. MO is a 100% natural product of the Mediterranean and it is suitable for human consumption as it is processed according to the legal standards. Previous research has shown that a total of 90 compounds have been identified in MO (50% monoterpene hydrocarbons, 20% oxygenated monoterpenes, 25% sesquiterpenes) [5]. Monoterpenes seem to exert favorable health effects by regulating mechanisms of oxidative stress and inflammation [6,7]. Additionally, previous studies have shown numerous valuable properties of these compounds such as antibiotic resistance modulation, antitumor, anticoagulant, antimicrobial, anti-Leishmania, antimalarial and analgesic effects [8,9,10].

We have previously investigated for the first time the kinetics and bioavailability of Mastiha’s main triterpenes in healthy subjects. Additionally, we have shown an antioxidant potential after acute consumption of Mastiha [11]. The aim of the present study was to investigate the absorption of the main monoterpenes of MO in humans for the first time, namely α-pinene (PubChem CID: 6654), myrcene (PubChem CID: 31253) and β-pinene (PubChem CID: 440967). To achieve this we employed a novel GC-MS-MS application, so that we overcome matrix difficulties with the tandem MS technique. Additionally, the effect on human antioxidant capacity was assessed with the serum oxidisabilty assay based on the existing data regarding the antioxidant properties of monoterpenes. 

## 2. Experimental methods

### 2.1. Materials

All monoterpene standards were purchased from Sigma-Aldrich (St. Louis, MO, USA) and were of high purity (>97%). All organic solvents were at least of LC grade and purchased from Merck KGaA, Darmstadt, Germany. MO was kindly donated by The Chios Gum Mastic Growers Association. MO comes out through distillation process with water and it is 100% natural. MO is resinous liquid, colourless to faintly yellow and has a balsamic, green and rustic odour. The density was 0.83 g/mL (20 °C). Its nutritional analysis is presented in Table S1.

### 2.2. Ethics

Informed consent was obtained by all eligible subjects before they participated in the study. Their rights to physical and mental integrity, to privacy and to the protection of the data were protected in accordance with the Data Protection Act 1998. The study was conducted in accordance with the Declaration of Helsinki and Tokyo for humans and the Principles of Good Clinical Practice. Harokopio University Ethics Committee (49/29-10-2015) approved the protocol. The trial was registered on ClinicalTrials.gov and was assigned with Identifier NCT04290312.

### 2.3. Study Design

An open label intervention trial was designed to assess the postprandial plasma levels of the main monoterpenes present in MO and their effect in antioxidant capacity. A kinetic approach was applied. Fifty apparently healthy male adults were screened. Ten volunteers were found eligible to the study according to specific criteria (Table 1). Eligible subjects were informed about the aims, methodology, expected effect and any risk from the Information Leaflet of the study, before signing an Informed Consent. The investigator explained that participation was voluntary and participants received a copy of the signed Informed Consent.

### 2.4. Postprandial Study Protocol

#### 2.4.1. Baseline Assessment

A medical and dietary assessment took place after enrollment. A blood sample was collected for complete blood count, fasting glucose, total cholesterol, triglycerides, low-density lipoprotein (LDL), high-density lipoprotein (HDL), urea, creatinine, serum glutamic oxaloacetic transaminase (SGOT), serum glutamic pyruvic transaminase (SGPT), alkaline phosphatase (ALP), serum gamma glutamyltransferase (γ-GT), lactate dehydrogenase (LDH) and total bilirubin measurement on an automatic biochemical analyzer (Cobas 8000 analyser, Roche Diagnostics GmbH). In addition, anthropometric indices were measured twice. Body weight was measured with a weighting scale (Seca) to the nearest 0.1 kg, while height was measured with a standard stadiometer (Tanita) to the nearest millimeter. Body Mass Index was calculated. In addition, body composition (fat mass, muscle mass, bone mass, total body water) was assessed with Bioelectric Impedance Analysis (Tanita, SA165 A-0950U-3).

#### 2.4.2. Intervention

**Figure 1:**
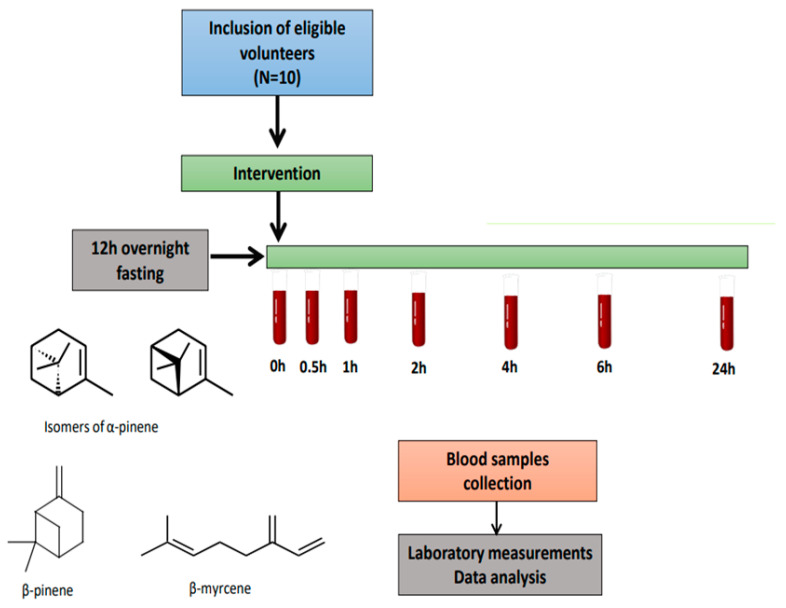
Study flowchart.

After overnight fasting, a plastic cannula was inserted in an arm vein of the volunteers in order discomfort during consecutive blood sampling to be minimized. A blood sample was obtained on time-point 0 h and after that the subjects consumed 1 mL of MO. This dose was selected based upon the study of Papada et al. [11] where healthy volunteers were administered with 10 g of Mastiha (containing ~10% MO). Afterwards, blood samples were collected on time-points 0.5 h, 1 h, 2 h, 4 h, 6 h and 24 h after MO consumption (Figure 1) and were centrifuged at 3000 rpm for 10 min at 4 °C for plasma and serum isolation. In this single-arm study all volunteers were assigned to the intake of monoterpenes present in MO. Measurements on time-point 0 h served as control, as these are considered known constants in absorption studies. All samples were stored at −80 °C until further analysis.

### 2.5. Analytical Techniques and Assays

#### 2.5.1. Detection and Quantification of Monoterpenes in Plasma Samples

A Thermo Ultra GC-Thermo XLS Quantum GC-MS-MS Triple Quad with a DB-5 ms 30 × 0.25 × 0.25 capillary column (Merck KGaA, Darmstadt, Germany) was used. The sample was injected using a Programmed Temperature Vaporizing (PTV) injector that allows concentration of samples, using an automated sampler. Injection volume was 1 µL.

To be able to determine low ppb levels, a GC-MS-MS technique was performed. Signal to noise improves radically compared to traditional GC-MS methods. Full scan MS-MS was first employed in analytical standards which run individually to determine the parent and daughter ions and retention times were validated with the use of analytical standards which run individually.

Gas Chromatograph conditions:

Initial temperature was held at 50 °C for 2 min. Then temperature was ramped to 60 °C with a rate of 5 °C/min. The temperature reached 270 °C at a rate of 7 °C/min. Injector temperature was set at 250 °C. The splitless injected volume was 1 µL. At 1.2 min the splitless valve switched.

The MS-MS conditions were as follows: Parent Ion was m/z 93 and daughter ions were 91 and 77 for all compounds. Collision energy was set at 8 V and collision gas pressure was 1.5 mTorr. MS-MS scan time was set at 0.15 s. The peak width was maintained at 0.7 amu.

For sample preparation, a 1:1 mixture of ethyl-acetate:hexane was used and equal volumes of blood plasma and extractant were put in centrifuge tubes and vortexed for 20 min. The samples were then centrifuged and filtrated before injection.

To be able to determine low ppb levels, a GC-MS-MS technique was performed, which, compared to traditional GC-MS methods, improves signal to noise values and thus sensitivity in the identification of compounds. Full scan MS-MS was first employed in analytical standards which run individually to determine the parent and daughter ions of the three terpenes and their retention times. Identification of chromatographic peaks was accomplished by using the parent ion and most abundant daughter ion of each compound and comparing the retention times with those of reference standards. For quantification, blank plasma samples (collected prior to MO administration) were spiked with standard monoterpene solutions (1–2000 μg/L), and were treated identically to the samples, since the standard addition method used eliminates the matrix effect. The standard monoterpene solutions were prepared in ethyl acetate in the range 2–2000 μg/L by diluting stock solutions (1 mg/mL). In all preliminary tests the average recovery for the monoterpenes studied was >80% and the detection limit was 0.02 µg/L.

#### 2.5.2. Kinetics of Monoterpenes in Plasma

The maximum concentrations of monoterpenes (Cmax) and the time intervals (Tmax) were obtained from each participant’s plasma concentration–time curve. The area under the plasma concentration–time curve (AUC) was analysed using the Linear Trapezoidal method.

#### 2.5.3. Antioxidant Capacity

The susceptibility of serum lipoproteins to oxidation induced by copper sulphate was estimated as previously described [12]. The produced conjugated dienic hydroperoxides were measured in an Elisa reader (Biotek PowerWave XS2). The increase in absorbance (245 nm) was plotted against time. The lag-time preceding oxidation was expressed in seconds (tLAG) according to Esterbauer and Jurgens [13]. All experiments were conducted at least in duplicate.

### 2.6. Statistics

Data were analysed with the Statistical Package for the Social Sciences (SPSS 21.0, SPSS Inc., Chicago, IL, USA). Non-parametric Friedman’s test was applied to evaluate differences in terpenes concentration and total serum oxidizability between the time-points. Level of statistical significance was set at *p* < 0.05. All data are presented as mean values ± standard error of mean (SEM). The G*Power 3 software (HHU, Dusseldorf-Germany, 2007) program was used to calculate the sufficient sample size with an αlpha-value of 5% and a power of 0.8. The sufficient sample size obtained was eight participants.

## 3. Results

All subjects completed the intervention. No adverse effects of MO intake were reported. Table 2 presents anthropometry and biochemical profile at baseline. Biochemical indices of hepatic and renal function were quantified to assess the safety of the dose administered (Table S2). As depicted in Table 2 and Table S2, all the parameters were within the normal range.

### 3.1. Profiling of MO

The total ion chromatogram (TIC) of extract from MO is presented in Figure S1. The main peaks in MO are respective to α-pinene (82.16%), myrcene (8.53%), and β-pinene (2.41%). Other phytochemicals detected in MO included linalool (0.84%), limonene (0.83%), camphene (0.64%), caryophyllene (0.46%), caryophyllene oxide (0.21%) and verbenone (0.20%).

### 3.2. GC-MS-MS Analysis of Plasma Samples

**Figure 2:**
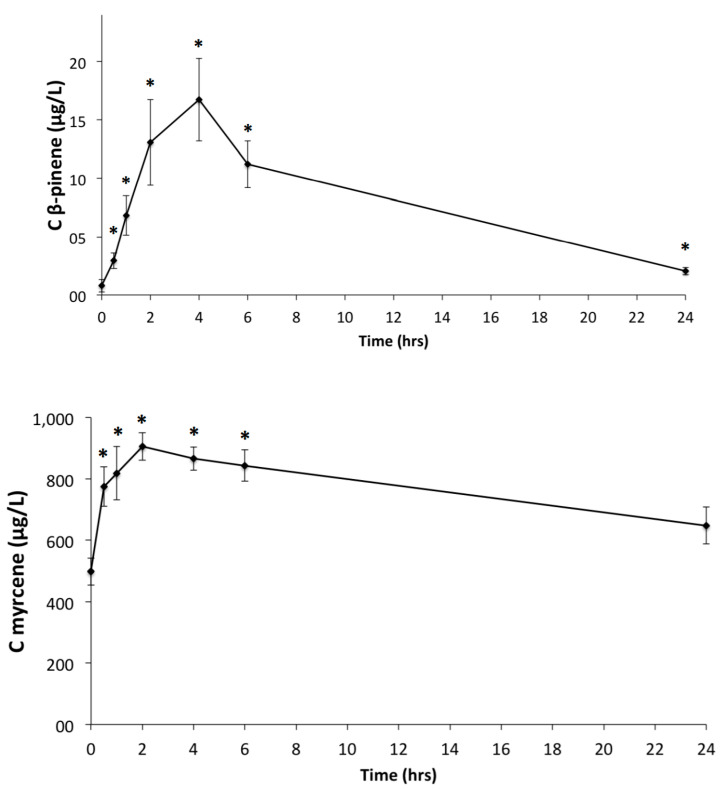

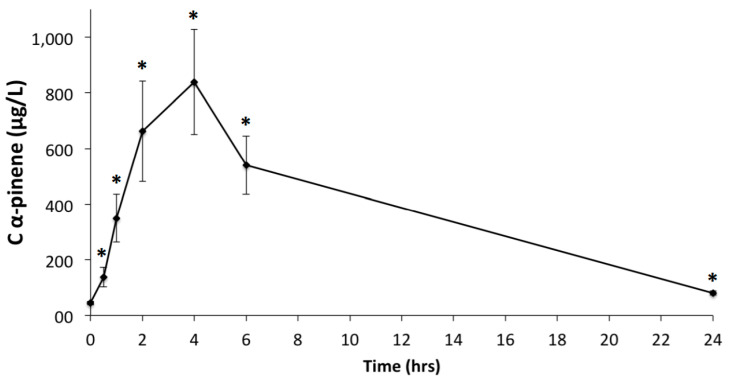
Plasma concentration-time curves for β-pinene, myrcene and a-pinene. Values are presented as mean ± standard error of mean. * *p* <0.05.

The major terpenes of MO, myrcene, α-pinene and β-pinene were detected in plasma samples applying in-house GC-MS-MS based method. A typical chromatogram of a plasma sample of a volunteer on time-point 4 h is depicted in Figure S2. The targeted monoterpenes could be detected already 0.5 h after MO consumption. Myrcene concentration at 0.5 h (774.8 ± 64.0 μg/L) increased significantly compared with baseline (*p* = 0.012), reached its peak at 2 h (905.6 ± 44.6 μg/L) and followed a decreasing trend until 24 h, when its concentration (648.2 ± 60.3 μg/L) was not significantly increased compared with baseline (*p* = 0.128). Alpha-pinene level followed a similar pattern but reached the peak at 4 h (839.1 ± 188.8 μg/L) with this concentration being significantly higher compared with baseline (*p* = 0.008). The level of α-pinene on timepoint 24 h (80.6 ± 7.3 μg/L) remained significantly increased compared with baseline (*p* = 0.028). The same pathway was followed for β-pinene. The peak concentration was observed at 4 h (16.7 ± 3.5 μg/L) and was significantly increased compared with baseline (*p* = 0.008). At 24 h the concentration (2.1 ± 0.3 μg/L) remained increased compared with baseline (*p* = 0.046). The mean plasma concentration-time curves are illustrated in Figure 2.

Table 3 depicts the plasma kinetic parameters, namely Cmax, Tmax and AUC. The highest Cmax was observed for myrcene (966.6 ± 89.7 μg/L), while the lowest Cmax was observed for β-pinene (18.0 ± 10.7 μg/L). The highest Tmax was 3.8 ± 1.2 h for α-pinene and the lowest was 2.2 ± 1.7 h for myrcene. The highest AUC was observed for myrcene and the lowest for β-pinene.

### 3.3. Antioxidant Capacity

**Figure 3:**
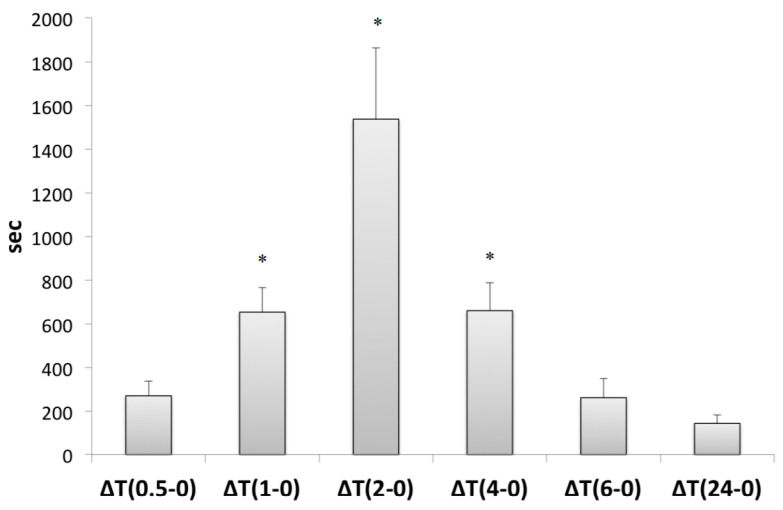
Antioxidant capacity was estimated applying the copper sulphate oxidation assay. * *p* < 0.05.

The antioxidant capacity is given as lag time (tLAG) in seconds, and particularly as the difference (ΔT) of tLAG of each time point from tLAG 0 h. Antioxidant capacity tended to increase since time point 0.5 h. This rise was significant on 1 h interval (653.2 ± 111.8 s), reached a peak on 2 h interval (1538.5 ± 327.2 s) and remained statistically significant until 4 h post-ingestion (660.1 ± 129.3 s) (*p* < 0.05) (Figure 3).

## 4. Discussion

Herein, we aimed at assessing whether humans absorb monoterpenes that are abundant in MO. Previous experiments on rats set the oral lethal dose—LD50—of MO at a dose of 5 g/kg body weight [14]. Our results show for the first time that MO consumption at the dose of 1 mL is safe, since there were no adverse effects and the parameters of renal and hepatic function were within the normal range after MO consumption. This finding is crucial, since natural products like MO could serve as potent weapons against pathological conditions related with oxidative stress and inflammation. This fact coincides with the postprandial kinetic study of Papada et al. with Mastiha at a dose of 10 g (containing approx. 1 mL MO) [11].

According to our knowledge this is the first study showing that the major terpenes of MO are bioavailable in human plasma using a method with a significant methodological advantage. To be able to determine low ppb levels in a complicated matrix like blood plasma, a GC-MS-MS technique was performed. Signal to noise improves significantly compared to traditional GC-MS methods.

Our results showed that myrcene, α-pinene and β-pinene are detected in plasma already after 0.5 h reaching their peaks at 2 h to 4 h post-ingestion. These time-points were slightly different; a study on rats showed that α-pinene was also bioavailable by 0.17 h reaching its peak concentration in plasma 2.5 h after oral administration [15]. However, it is important to mention that human and animal metabolism share not only common but also different metabolic characteristics that do not allow us direct comparisons and generalizations. As regards to our previous study in humans investigating the absorption of triterpenes, we have shown similar patterns of kinetics. The main triterpenes of Mastiha were detected 0.5 h after consumption and reached their peak concentration between 2 h and 4 h. These patterns coincided with the present study but cautious interpretation is necessary, since Mastiha has a different matrix compared to MO and additionally triterpenes are much more complex compounds than monoterpenes [11]. The fact that monoterpenes of MO reach the plasma unaltered could partially explain the beneficial properties for human health that have been attributed to MO since antiquity.

Herein the retention time of b-pinene was found lower to the respective of myrcene. This result is in compliance with the study of Thao et al. [16] using a similar DB5 column. Overall, when the natural product was profiled, more terpenes were detected compared to the blood samples. These results are of no surprise since metabolism of terpenes is quite complex. Different pH conditions, mechanical and enzymatic activities, and also transformations into generally more water-soluble and more readily excreted in the urine compounds take place. These changes are observed primarily in the liver, gastrointestinal tissue, lungs, kidneys, brain and blood [17].

As regards oxidative stress, we evaluated serum resistance to oxidation, which was significantly increased on time point 1 h, reached a peak on time point 2 h and remained statistically significant until 4 h post-ingestion. In our previous study on the absorption of triterpenes that are abundant in Mastiha, serum resistance to oxidation increased significantly at 4 h, reached its peak at 6 h and remained significantly at 24 h. This effect was associated with the availability of the detected triterpenes in plasma. Most studies evaluating the absorption of phytochemicals have reported a peak in serum antioxidant capacity 1–2 h after ingestion, a finding that comes into agreement with our results. A postprandial study of Kanellos and coworkers assessed the absorption and plasma kinetics of phytochemicals present in raisins and their effect on serum oxidation resistance in healthy humans [18]. Serum resistance to oxidation and plasma total phenolics reached their peak 1 h after raisin consumption. Despite the similar patterns revealed, the different matrices of the foods been studied should not allow us to make direct comparisons.

It is also important to mention that the terpenes detected and quantified in plasma could affect directly oxidative stress, but also endogenous antioxidants may be implicated in the increase of resistance to oxidation. However, we could hypothesize that the differences in antioxidant capacity could be attributed to terpenes that have proven antioxidant properties [19]. A study on the essential oil from black pepper that contains myrcene, α-pinene and β-pinene pointed out antioxidant properties in vitro [20]. More specifically, the essential oil scavenged diphenyl-2 picrylhydrazyl (DPPH), nitric oxide (NO), 2,2′-azino-bis(3-ethylbenzthiazoline-6-sulphonic acid)ABTS and chelated Fe^2+^. Although further research is necessary to investigate the direct effects of monoterpenes on antioxidant defense mechanisms in humans, our results could point towards this direction.

Our protocol has significant strengths. Gender differences, the menopause and menstrual cycle with hormonal fluctuations in females seem to affect the absorption of phytochemicals [21,22]. Thus, the recruitment of males is one of our protocol’s advantages so that the kinetics of the monoterpenes is not affected by those factors. Additionally, strict adherence to the inclusion and exclusion criteria during recruitment of participants led to a homogenous sample. An additional strength is the application of a GC-MS-MS technique not previously described in the monoterpene detection in human plasma. This application allows for the identification and quantification of these aromatic phytochemicals even at low detection levels without the matrix interferences.

## 5. Conclusions

This is the first study showing that myrcene, alpha- and beta pinenes abundant in MO and several natural products are bioavailable in plasma already 30 min after consumption of a single dose. Additionally, they may contribute to antioxidant defense since serum resistance to oxidation increased 1 h after their administration. Monoterpenes exhibit several beneficial effects and our findings could contribute to understanding their usage and applications on human health. Proving that monoterpenes in MO are bioavailable in human plasma is the first significant step towards the usage of foods and natural products for medicinal purposes. Further research is necessary to characterize the kinetics of these monoterpenes in human metabolism.

## Figures and Tables

**Table 1:** Inclusion and exclusion criteria.

Inclusion Criteria	Exclusion Criteria
Sex: Male	Obesity
Age: 20–40 years old	Alcohol or drug abuse
	Medication, vitamins and inorganic supplements
	Vegan and macrobiotic diet before and during the trial
	Gastrointestinal diseases (i.e., atrophic gastritis, Inflammatory Bowel Disease, peptic ulcer, gastrointestinal cancer)

**Table 2:** Baseline characteristics.

Anthropometric	
Age (year)	25.8 ± 2.4
Height (cm)	178.0 ± 1.7
Weight (kg)	85.2 ±3.7
BMI (kg/m^2^)	26.9 ± 1.2
Body fat (%)	19.9 ± 2.9
Total Body Water (%)	57.1 ± 2.0
Muscle mass (kg)	64.2 ± 1.4
Bone mass (kg)	3.3 ± 0.0
Biochemical	
Red Blood Cells (/μL)	5,256,250.0 ± 111,624.9
White Blood Cells (/μL)	6923.8 ± 646.6
Hemoglobin (g/dL)	16.1 ± 0.4
Hematocrit (g/dL)	46.1 ± 1.0
Mean Corpuscular Volume (fl)	87.8 ± 1.1
Mean Corpuscular Hemoglobin Concentration (g/dL)	35.0 ± 0.3
Mean Corpuscular Hemoglobin (pg/RBC)	30.7 ± 0.3
Glucose (mg/dL)	89.5 ± 3.4
Total Cholesterol (mg/dL)	182.4 ± 11.6
Triglycerides (mg/dL)	86.7 ± 16.4
High-Density Lipoprotein (mg/dL)	45.3 ± 2.5
Low-Density Lipoprotein (mg/dL)	111.7 ± 7.4
High Risk Rate	4.0 ± 0.4

Values are Mean ± Standard Error of Mean.

**Table 3:** Kinetics of Myrcene, α-Pinene and β-Pinene in plasma.

Terpenes	C_max_ (μg/L)	T_max_ (h)	Area Under Curve (μg·h/L)
Myrcene	966.6 ± 89.7	2.2 ± 1.7	15318.0 ± 7313.3
α-Pinene	914.8 ± 551.2	3.8 ± 1.2	7865.2 ± 5547.3
β-Pinene	18.0 ± 10.7	3.6 ± 0.9	164.0 ± 110.8

Values are Mean ± Standard Error of Mean.

## Supplementary Materials

The following are available online at https://www.mdpi.com/2304-8158/9/8/1019/s1, Table S1: Nutritional analysis of MO, Table S2: Biochemical parameters of hepatic and renal function, Figure S1: The total ion chromatogram (TIC) of extract from MO, Figure S2: Chromatogram of a plasma sample on time point 4 h.
